# Adherence to antiretroviral therapy among female sex workers in Kampala, Uganda

**DOI:** 10.4102/jphia.v16i1.1376

**Published:** 2025-12-18

**Authors:** Dennis M. Ssemakula, Sheila N. Balinda, Yunia Mayanja, Onesmus Kamacooko, Andrew Abaasa, Janet Seeley

**Affiliations:** 1 Department of Epidemiology, Medical Research Council/Uganda Virus Research Institute and London School of Hygiene and Tropical Medicine Uganda Research Unit, Entebbe, Uganda; 2 International Traineeships in AIDS Prevention Studies (I-TAPS) Program, University of California, San Francisco I-TAPS, San Francisco, United States; 3 Department of Vaccine Theme and Viral Pathogens, Medical Research Council/Uganda Virus Research Institute and London School of Hygiene and Tropical Medicine Uganda Research Unit, Entebbe, Uganda; 4 Department of Infection Biology, London School of Hygiene and Tropical Medicine, London, United Kingdom; 5 International Traineeships in AIDS Prevention Studies (I-TAPS) Program, University of California, San Francisco I-TAPS, San Fracisco, United States; 6 Department of Statistics and Data, Medical Research Council/Uganda Virus Research Institute and London School of Hygiene and Tropical Medicine Uganda Research Unit, Entebbe, Uganda; 7 Department of Statistics and Data, The Medical Research Council/Uganda Virus Research Institute and London School of Hygiene and Tropical Medicine Uganda Research Unit, Entebbe, Uganda; 8 Department of Global Health and Development, London School of Hygiene and Tropical Medicine, London, United Kingdom

**Keywords:** ART, adherence, female sex workers, information and communication technology

## Abstract

**Background:**

Increased access to antiretroviral therapy (ART) for key populations who bear a disproportionate burden of human immunodeficiency virus (HIV) and acquired immunodeficiency syndrome (AIDS), including female sex workers (FSWs), reduces onwards transmission. This is, however, dependent on achieving high levels of adherence to ART.

**Aim:**

To determine the level of adherence to ART and associated factors among FSWs.

**Setting:**

An urban HIV clinic in Kampala, Uganda.

**Methods:**

This cross-sectional study enrolled 226 FSWs accessing HIV care between May 2017 and June 2017. We assessed self-reported adherence using interviewer-administered questionnaires and reviewing medical records. We defined high-level adherence as those who scored ≥ 95% at assessment. Using multivariable logistic regression, we identified factors independently associated with adherence.

**Results:**

Overall, 59.2% of participants were adherent to ART. Major reasons for non-adherence were being away from home (40.8%) and forgetfulness (26.7%). In the multivariable model, owning a phone (adjusted odds ratios [AOR]: 2.90; 95% confidence intervals [CI]: 1.07, 7.88), a 10-year increase in age (AOR: 1.60; 95% CI: 1.00, 2.60) and being a widow (AOR: 0.22; 95% CI: 0.05, 0.87) were independently associated with adherence.

**Conclusion:**

This baseline assessment builds a case for the development and scale-up of targeted intervention strategies to increase ART adherence among FSWs. Incorporating information and communication technology in routine adherence counselling could be scaled up among FSWs.

**Contribution:**

Our study highlights the possibility of integrating mobile phone-based adherence support in routine HIV care and informs the design of targeted interventions to curtail HIV transmission.

## Introduction

The human immunodeficiency virus (HIV) and acquired immunodeficiency syndrome (AIDS) remains a public health problem world over, with 38 million infected and 690 000 deaths reported in 2021.^1^ Key populations including and their sexual partners are estimated to have accounted for 62% of new HIV infections globally in 2019.^1^ These include men who have sex with men (MSM), people who inject drugs (PWID) and female sex workers (FSWs). Specifically, FSWs globally continue to share a disproportionate burden of HIV with an estimated 30 times higher risk of acquiring infection than adults aged 15–49 years in the general population.^1^ In sub-Saharan Africa (SSA), a systematic review of studies conducted in 25 countries indicated that 36.9% of FSWs were HIV-positive, with HIV prevalence as high as 70.7%^2^ in some countries. In Uganda, HIV prevalence among FSWs ranges from 32.4% to 52%^3,4^ compared to 9.5% among women in the general population.^5^ Female sex workers and their clients are estimated to contribute 18% of the new infections in Uganda.^5^

Several factors influence the higher risk of HIV acquisition and thus the high prevalence among FSWs. These include alcohol abuse, multiple clients and other sexual partners, inconsistent condom use and a higher prevalence of sexually transmitted infections (STIs).^4,6^ Access to HIV preventive services is limited because of lack of community-based support systems, stigmatisation and criminalisation, high mobility and the judgemental attitude of some health workers towards FSWs.^7,8,9,10^ In addition, FSWs with high mobility may face additional transport challenges to health facilities.^7^

Female sex workers are prioritised for increased access to antiretroviral therapy (ART), to improve their health outcomes and prevent onward transmission of HIV to sex partners. Since 2013, Uganda has been implementing ‘test and treat’ for key populations, including FSWs,^11^, which developed into universal ART for all, following World Health Organization (WHO) guidelines.^12^ However, to optimise full benefits of ART, high-level adherence sustains viral suppression, reduces risk of drug resistance and onward HIV transmission.^13,14^ Adherence is defined as the patient’s ability to follow a treatment plan by taking the right medicine, in the right dose, at the right time and in the right way. Over 95% adherence to ART confers optimal therapeutic effectiveness.^15^ Factors that influence adherence to ART include patient-related factors, such as alcohol and other substance abuse,^16^ age, sex, level of education, stigma, social support and HIV status disclosure.^17,18^ The treatment-related factors are side effects, pill burden and severity of disease.^19^ In addition, factors related to the patient–provider relationship, like trust and acceptance^20^ and the healthcare system, also affect adherence.^21^

Female sex workers face unique realities that determine their contact with the healthcare system. For instance, a study in Kampala found that only 45% of HIV-positive FSWs knew their HIV sero-status, of whom only 37.8% reported being on ART and only 35.2% achieving viral suppression.^22^ Studies have attributed low ART uptake and interrupted engagement in HIV care among FSWs to their high mobility,^23^ an environment of punitive laws and discrimination against FSWs, stigma, judgemental attitude from some health workers, frequent arrests and intimate partner violence.^9,24^ Furthermore, despite policies to increase access to ART among FSWs, appreciation of their unique realities of life and efforts to improve their low agency in the fight against HIV, there is still a paucity of information regarding adherence to ART and its associated factors for FSWs in SSA. One systematic review largely from industrialised countries and another from India indicate that adherence to ART in FSWs ranges between 48%^25^ and 76%.^26^ We build on this work with HIV-positive FSWs accessing ART from an urban clinic in Kampala Uganda to determine the level of ART adherence and associated factors. Our study assessed adherence to ART and associated factors among HIV-positive FSWs attending an urban clinic in Uganda.

## Research methods and design

### Study design and setting

We conducted a cross-sectional study between May 2017 and June 2017. The Good Health for Women Project (GHWP) is in Mengo Kisenyi, a densely populated slum in the central division of Kampala, the capital city of Uganda. The GHWP clinic was established in 2008 by Medical Research Council/Uganda Virus Research Institute (MRC/UVRI) in collaboration with the Uganda Ministry of Health. The GHWP was set up to study the epidemiology of HIV and other STIs among FSWs, and to provide free enhanced HIV prevention services to this key population. These include treatment of common illnesses, comprehensive HIV care, management of other STIs, reproductive health services, condom promotion and distribution and alcohol risk reduction counselling. These services are delivered at 3-monthly visits and according to the clients’ demand. Although GHWP started offering ART in 2013, ‘test and treat’ began in August 2014; a government of Uganda policy where HIV-positive FSWs start ART immediately irrespective of cluster of differentiation 4 (CD4) count.^11^

### Study participants and eligibility criteria

Good Health for Women Project patients who are FSWs are defined as women who engage in sex with men for money and other favours. Female sex workers are recruited to the clinic at night through a peer-led approach from their operating hotspots (e.g. bars, brothels, nightclubs, streets, eating places, lodges and guesthouses). Greater details on the recruitment of FSWs to the GHWP clinic are described elsewhere.^4^ Eligibility criteria were being 18 years old and above, accessing ART from GHWP and having been on ART for at least 6 months. Participants were enrolled consecutively as they came either for their routine clinic visits or for any other reason.

### Antiretroviral therapy preparation, initiation and follow-up

Prior to initiating ART, baseline CD4 counts and pre-ART adherence counselling was performed on confirmed HIV-positive clients. Antiretroviral therapy was dispensed first for 14 days, and then 1-monthly, 2-monthly, and 3-monthly refills were given only when the client was determined to be stable. A fixed-dose combination of tenofovir disoproxil fumarate (TDF), lamivudine (3TC) and efavirenz (EFV) is used as the first-line regimen, unless otherwise indicated. This was a single pill (TRUVADA) used once a day by adults. For follow-up, viral load testing was performed 6 months after ART initiation, repeated yearly if viral suppression had been achieved, and, if not, 4–6 months following Uganda Ministry of Health Guidelines.^27^ Ongoing adherence counselling was provided for the clients at every clinic visit as appropriate. Specifically, those clients for whom viral suppression was not achieved, at least three sessions of intensive adherence counselling were performed monthly by the nurse counsellor until viral suppression was achieved. Screening for and management of opportunistic infections and other STI was performed at every contact with the clients.

### Study variables, measures and data collection

#### Study outcome

We categorised the outcome variable, ART adherence, as a binary adherence or non-adherence. Adherence was measured using self-reports of missed pills over a 30-day recall period. Studies have shown that self-reported data correlate with viral load changes.^28,29,30^ Participants who reported taking ≥ 95% of prescribed pills were considered adherent and those who took < 95% of the pills were considered non-adherent.

#### Exposure variables

Potential predictors of ART adherence were categorised into social demographics, those related to disclosure, behavioural variables and treatment-related clinical variables. Social demographics variables included age, highest level of education attained, marital status, working in a bar or lodge and owning a phone. Those related to disclosure included: HIV status and ART disclosure to either any significant other or to male regular sexual partner. Social support was defined as a participant having a treatment companion and not living alone. Stigma was defined as the participant never disclosed her HIV status or did not want clinic staff to visit her home for fear of being discriminated against by her neighbours. Behavioural variables included mobility and alcohol use in the last 3 months, as Yes or No answers. Mobility was considered as staying at the current location less than 50% of her time or having changed place of residence at least once in the previous 1 year. Treatment-related clinical variables included duration from HIV diagnosis to ART initiation, duration on ART, baseline CD4 count, possession of a medicine reminder tool, possession of an ART adherence card, WHO stage, pregnancy at ART initiation, type of regimen, history of concomitant medications in the past 1 month and viral suppression defined as viral load of less than 1000 copies/mL. The most recent test results from the patients’ medical records were preferred to real-time testing to meet study costs.

### Data collection and laboratory methods

Our trained study staff collected data using pre-tested interviewer-administered tools and data extraction from participant files. Pretesting of the data collection tool was performed in the first week of data collection to determine suitability, face validity, comprehension, and to achieve a common understanding of the tool among the nurse counsellors. Quality control checks were performed for each completed case report form to ensure completeness, accuracy and correctness.

As part of routine clinical care, blood samples were collected to assess HIV viral load and CD4+ T-cell count. Baseline CD4 + T-cell counts were performed on ART initiation and viral load (VL) was performed at least 6 months from ART initiation. Baseline CD4 + T-cell counts were performed on plasma using Multi-test Trucount tube CD4, CD8 and CD3-Lyse no wash (Becton D Biosciences, United States). Viral load testing was performed on plasma using an Abbott Real-time HIV-1 PCR assay (Abbott Park, Illinois, US) from the Uganda Central Public Health Laboratories.

### Statistical analyses

We entered data into epi-data, cleaned and exported to STATA 15.0 (StataCorp, College Station, TX, United States) for analysis. We categorised and summarised participants’ characteristics by counts and percentages. We analysed continuous variables using appropriate measures of central tendency, means or medians, and their relevant measures of dispersion, standard deviations and interquartile ranges, respectively. We determined the proportion of non-adherent FSWs who scored ≥ 95% at assessment divided by the total number included in the analysis and expressed as a percentage. We used logistic regression to identify factors associated with adherence at bivariate analysis. We selected variables with statistical significance *p* < 0.1 on log likelihood ratio tests (LRT) for multivariable logistic regression. We maintained ‘alcohol use’ variable in the final model because literature shows that it is a strong predictor of ART adherence. We presented results adjusted odds ratios (AOR) with *p*-values and 95% confidence intervals (CI). The *p*-values < 0.05 were considered significant.

### Ethical considerations

Ethical clearance to conduct this study was obtained from Uganda Virus Research Institute Research Ethics Committee (No. MRCU/07/815) and Uganda National Council for Science and Technology (No. HS 364). Our study presented more than minimal risks including potential unintended disclosure of HIV and FSW status to the community during home visits. Noteworthy, we performed a secondary analysis of clinical information without any invasive procedures that diminished some risks. Furthermore, we obtained written informed consent from all participants, and all data were de-identified prior to analysis.

## Results

**FIGURE 1:**
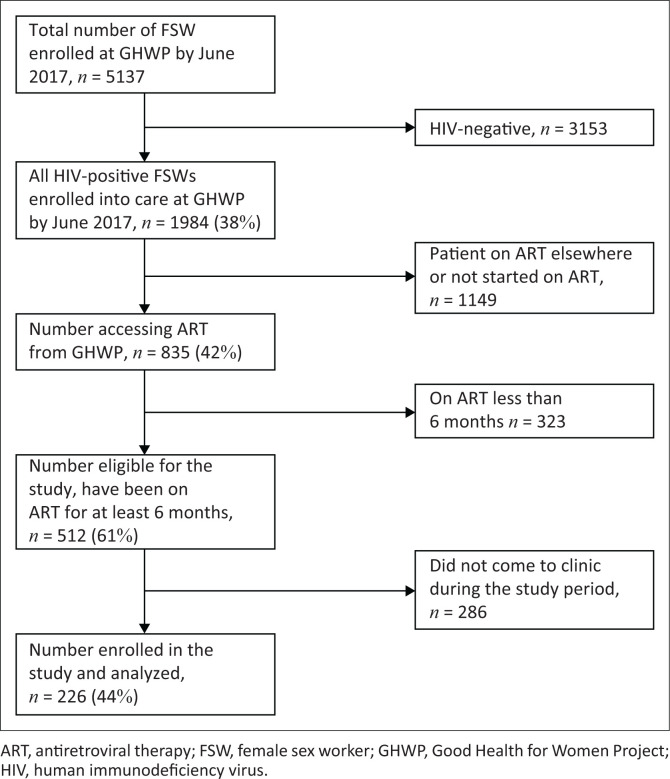
Flow diagram of female sex workers recruited for a study of antiretroviral therapy adherence, Kampala, Uganda, 2017.

A total of 5157 FSWs had been cumulatively recruited into the GHWP by the end of June 2017 (Figure 1). About 2 in 5, 38.5% (*n* = 1984) were HIV-positive, of whom 42.1% (*n* = 835) accessed ART from the GHWP clinic. The remainder (*n* = 1149) had either not yet started ART or were receiving ART elsewhere. Among those accessing ART from GHWP, 512 participants (61.3%) were on ART for more than 6 months at GHWP and were eligible for the study, of whom 226 (44.1%) were included in the final analysis of this study. It is noteworthy that, we excluded the 38.7% (*n* = 323) on ART for less than 6 months.

### Characteristics of study participants

The FSWs had a mean age of study participants of 32 years (standard deviation [s.d.]: ±6.5), with 56.2% falling within the 25–35 years age bracket. A total of 56.6% had attained at least primary-level education. While only 37.6% were separated or divorced, most FSWs (88.5%) reported disclosing their HIV status to a significant other (Table 1). Over half (56.2%) reported consuming alcohol over the last 3 months. The majority (90.3%) reported owning a mobile phone.

**TABLE 1:** Baseline characteristics of a sample of human immunodeficiency virus (HIV)-positive female sex workers (*n* = 226) attending clinic at Good Health for Women Project, Kampala, Uganda, assessed for adherence to antiretroviral therapy between the months of May and June 2017 (*n* = 226).

Variable	Mean	s.d.	Median	Range	IQR	*n*	Col %
Age (years)	32	−6.5	-	-	-	-	-
**Age group (years)**							
< 25	-	-	-	-	-	30	13.3
25–35	-	-	-	-	-	127	56.2
> 35	-	-	-	-	-	69	30.5
**Level of education**							
None	-	-	-	-	-	25	11.1
Primary	-	-	-	-	-	128	56.6
Secondary and beyond	-	-	-	-	-	73	32.3
**Marital status**							
Married	-	-	-	-	-	55	24.3
Widowed	-	-	-	-	-	12	5.3
Separated or divorced	-	-	-	-	-	85	37.6
Never married	-	-	-	-	-	74	32.7
**Social support†**							
No	-	-	-	-	-	62	27.4
Yes	-	-	-	-	-	164	72.6
**Stigma** ‡							
No	-	-	-	-	-	126	55.8
Yes	-	-	-	-	-	100	44.2
**HIV status disclosure to significant other**							
No	-	-	-	-	-	26	11.5
Yes	-	-	-	-	-	200	88.5
**HIV status disclosure to regular sexual male partner (among those with regular partner, *n* = 162)**							
No	-	-	-	-	-	92	40.7
Yes	-	-	-	-	-	70	31.0
**ART disclosure to any significant other**							
No	-	-	-	-	-	28	12.4
Yes	-	-	-	-	-	198	87.6
**Mobility§**							
No	-	-	-	-	-	158	69.9
Yes	-	-	-	-	-	68	30.1
**Works in bar or lodge**							
No	-	-	-	-	-	140	61.9
Yes	-	-	-	-	-	86	38.1
**Alcohol use in last 3 months**							
No	-	-	-	-	-	99	43.8
Yes	-	-	-	-	-	127	56.2
**Owns a phone**							
No	-	-	-	-	-	22	9.7
Yes	-	-	-	-	-	204	90.3
Duration in months from HIV diagnosis to ART initiation	0	−68	6	-	-	-	-
Duration of treatment in months	22	8	-	-	-	-	-
Baseline CD4 count	-	-	532	-	390–692	-	-
**Medicine reminder tool**							
No	-	-	-	-	-	49	21.7
Yes	-	-	-	-	-	177	78.3
**Adherence diary card**							
No	-	-	-	-	-	85	37.6
Yes	-	-	-	-	-	141	62.4
**WHO staging**							
Stage I	-	-	-	-	-	198	87.6
Stage II	-	-	-	-	-	20	8.8
Stage III	-	-	-	-	-	8	3.5
**Baseline CD4 counts (cells/µL)**							
< 350	-	-	-	-	-	45	19.9
350 – < 500	-	-	-	-	-	53	23.5
> 500	-	-	-	-	-	128	56.6
**Viral suppression**							
≥ 1000 copies/mL	-	-	-	-	-	14	6.2
< 1000 copies/mL	-	-	-	-	-	202	89.4
Missing	-	-	-	-	-	10	4.4
**Pregnant at ART initiation**							
No	-	-	-	-	-	206	91.2
Yes	-	-	-	-	-	20	8.8
**Type of regimen**							
TDF/3TC/ATV	-	-	-	-	-	3	1.3
TDF/3TC/EFV	-	-	-	-	-	219	96.9
TDF/3TC/LPV	-	-	-	-	-	1	0.4
TDF/3TC/NVP	-	-	-	-	-	3	1.3
**History of concomitant medications in last month**							
No	-	-	-	-	-	196	86.7
Yes	-	-	-	-	-	30	13.3

ART, antiretroviral therapy, WHO, World Health Organisation, s.d., standard deviation, IQR, interquartile range; HIV, human immunodeficiency virus; TDF, tenofovir disoproxil fumarate; 3TC, lamivudine; ATV, atazanavir; EFV, efavirenz; LPV, lopinavir; NVP, nevirapine; Col %, column percentage.

† , Considered if a participant reported having a treatment companion and does not stay alone.

‡ , Considered if a participant had never disclosed her HIV status and declined home visit by the clinic staff for fear of being suspected by the neighbours.

§ , Considered if a participant reported staying at the current location less than 50% of her time and had changed her place of residence at least once in the previous 1 year.

Clinically, most FSWs (87.6%) had initiated ART early at WHO stage 1 and had a mean duration on ART of 22 months (s.d.: ±8). Half FSWs had a baseline CD4+ T-cell count above 532 cells/mm^3^ (interquartile range [IQR]: 390–692). The majority (96.9%) of participants were on first-line regimen (TDF, 3TC and EFV). Viral suppression (< 1000 copies/mL) was observed in 89.4% of participants.

### Adherence and associated factors

**FIGURE 2:**
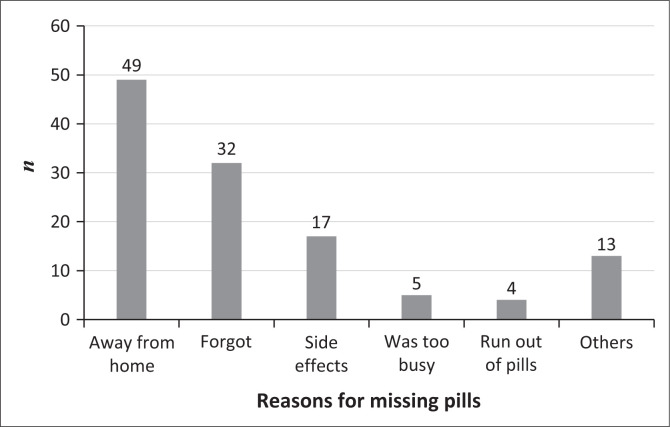
Reasons for missing by female sex workers (*n* = 120) assessed for adherence, Kampala, Uganda 2017.

Overall, 59.2% of participants were adherent to ART. Being away from home was the most important reason for missing pills, 40.8% (*n* = 120) (Figure 2). In the bivariate analysis, adherence to ART was more likely among participants who had social support compared to those who did not (odds ratio [OR]: 1.85; 95% CI: 1.02, 3.34; *p* = 0.041), those who owned a phone at the time of the study (OR: 3.53; 95% CI: 1.38, 9.05; *p* = 0.006) and those who reported possession of a medicine reminder tool (OR: 2.35; 95% CI: 1.23, 4.40; *p* = 0.009). A 10-year increase in age of the client was associated with increased odds of adherence (OR: 1.59; 95% CI: 1.03, 2.43; *p* = 0.031).

In multivariable analysis, participants who owned a phone at the time of study were more likely to adhere to ART than those who did not (AOR: 2.90; 95% CI: 1.07, 7.88) (Table 2). There was an increased odds of adherence to ART for each 10-year increase in age (AOR 1.60; 95% CI: 1.00, 2.60). Compared to married women, widowed participants were less likely to adhere to ART (AOR: 0.22; 95% CI: 0.05, 0.87). The finding on alcohol use may have suffered social desirability bias from, especially, among drinkers of alcohol.

**TABLE 2:** Multivariable regression of adherence to antiretroviral therapy and associated factors among human immunodeficiency virus (HIV)-positive female sex workers attending clinic at Good Health for Women Project, Kampala, Uganda between May and June 2017 (*n* = 226).

Variable	Proportion adherent		Unadjusted		LRT (*p*-value)	Adjusted		*p*-value
	*n*	Col %	OR	95% CI		OR	95% CI	
**Overall**	134	59.2	-	-	-	-	-	-
**Age (per 10-year increase)**	33	28–37	1.59	1.03, 2.43	0.031	1.60	1.00, 2.60	0.047
**Level of education**							
None	11	44.0	1	-	0.261	-	-	-
Primary	78	60.9	1.99	0.84, 4.72	-	-	-	-
Secondary and beyond	45	61.6	2.05	0.82, 5.13	-	-	-	-
**Marital status**								
Married	37	67.3	1	-	0.084	1	-	-
Widowed	5	41.7	0.35	0.10, 1.25	-	0.22	0.05, 0.87	0.031
Separated or divorced	55	64.7	0.89	0.44, 1.83	-	0.77	0.35, 1.69	0.514
Never married	37	50.0	0.49	0.24, 1.00	-	0.60	0.27, 1.32	0.206
**Social support**								
No	30	48.4	1	-	0.041	1	-	-
Yes	104	63.4	1.85	1.02, 3.34	-	1.49	0.78, 2.84	0.228
**Stigma**								
No	79	62.7	1	-	0.242	-	-	-
Yes	55	55.0	0.73	0.43, 1.24	-	-	-	-
**HIV status disclosure to any significant other**								
No	14	53.8	1	-	0.55	-	-	-
Yes	120	60.0	1.29	0.57, 2.92	-	-	-	-
**HIV status disclosure to regular male sexual partner**								
No	53	57.6	1	-	0.293	-	-	-
Yes	46	65.7	1.41	0.74, 2.69	-	-	-	-
**ART disclosure**								
No	15	53.6	1	-	0.512	-	-	-
Yes	119	60.1	1.31	0.59, 2.89	-	-	-	-
**Mobility**								
No	96	60.8	1	-	0.495	-	-	-
Yes	38	55.9	0.82	0.46, 1.45	-	-	-	-
**Works in bar or lodge**								
No	83	59.3	1	-	0.998	-	-	-
Yes	51	59.3	1.00	0.58, 1.72	-	-	-	-
**Alcohol use in last 3 months**								
No	63	63.6	1	-	0.240	1	-	0.387
Yes	71	55.9	0.72	0.42, 1.24	-	0.77	0.42, 1.39	-
**Owns a phone**								
No	7	31.8	1	-	0.006	1	-	0.036
Yes	127	62.3	3.53	1.38, 9.05	-	2.9	1.07, 7.88	-
**Medicine reminder tool**								
No	21	42.9	1	-	0.009	1	-	0.108
Yes	113	63.8	2.35	1.23, 4.4	-	1.76	0.88, 3.51	-
**Adherence diary card**								
No	49	57.6	1	-	0.696	-	-	-
Yes	85	60.3	1.12	0.65, 1.93	-	-	-	-
**WHO staging**								
Stage I	117	59.1	1	-	0.756	-	-	-
Stage II	13	65.0	1.29	0.49, 3.36	-	-	-	-
Stage III	4	50.0	0.69	0.17, 2.85	-	-	-	-
**Baseline CD4 counts (cells/µL)**								
< 350	26	57.8	1	-	0.842	-	-	-
350–500	30	56.6	1.10	0.42, 2.91	-	-	-	-
> 500	78	60.9	1.32	0.55, 3.18	-	-	-	-
**History of concomitant medications in last month**								
No	114	58.2	1	-	0.379	-	-	-
Yes	20	66.7	1.44	0.64, 3.24	-	-	-	-

OR, odds ratio; CI, confidence interval; LRT, likelihood ratio test; HIV, human immunodeficiency virus; ART, antiretroviral therapy; WHO, World Health Organization; Col %, column percentage.

## Discussion

### Key findings

Firstly, our study discovered that adherence to ART was suboptimal among FSWs in the GHWP, with only 3 out of 5 attaining ≥ 95% level. Secondly, adherence to ART was independently associated with ownership of a mobile phone, older age by 10-year bands and marital status. Specifically, widows were less adherent to ART. Alcohol use was not associated with adherence in this key population.

### Strengths and limitations

Although our study had some strengths, these findings are better understood with regard to its limitations. We had a relatively large sample size of FSWs and conducted this study in a typical urban slum setting for FSW providing for generalisable findings. However, selection bias could have occurred as we enrolled only those FSWs who came to the clinic for their visits. As a result, we may have overestimated adherence to ART among the participants. Firstly, our measurement of adherence to ART was based on patients’ self-reports of missed pills, which are subject to recall and social desirability biases, potentially resulting in overestimated adherence. Good enough, self-reported adherence correlates well with viral load changes, is cheaper and more practical in low-income settings.^28,29,30^ A recent article that compared adherence to ART by appointment keeping, 7-day recall versus 30-day recall and found that only 30-day self-report measure was associated with a lower risk of virological failure (VF) (adjusted hazard ratio (aHR) = 0.14, 95% CI: 0.05–0.37).^31^ Another older study compared 3-day caregiver recall, 30-day visual analogue scale (VAS), Medication Event Monitoring System (MEMS), unannounced pill counts (UPC) and liquid formulation weights were collected monthly over a 1-year period. This study reported that 3-day caregiver recall; VAS and MEMS were found to be positively associated with increasing Nevirapine (NVP) concentration in hair, although associations were not statistically significant.^32^ However, recall approaches to measuring adherence are not without limitations. For example, a study on patient health literacy and adherence to ART demonstrated that overall, 57% of patients reported a 30-day recall of 100% adherence.^33^ Finally, we opted for the most recent viral load results instead of real-time viral load tests that could have overestimated adherence. Given our cross-sectional design, we recognise the limitations of correlating self-reported results with patient important outcomes.

### Interpretation in relation to existing evidence

Adherence among FSWs was relatively low compared to findings from a systematic review of studies largely from higher-income countries at 76%.^26^ Evidence from other studies reported FSW adherence to ART at 67%^34^ and 81%.^35^ In addition, our study reported lower adherence to ART compared to the general populations in SSA.^18,36^ Low proportions of ART adherence among FSWs have also been reported in India, in a study that estimated adherence at 48% using a combined measure of pill counts and self-reports.^25^

The major reason for missing pills by participants in our study was being away from home. Most FSWs do not usually operate from their areas of residence, where they are known, but rather go to other hot spots within the city where they are less likely to be known. It is possible that sometimes they either forget carrying their pills with them or spend more days away from home than they had anticipated.

We found a strong association between owning a mobile phone and adherence. This is consistent with the fact that most of the study participants who reported owning a mobile phone used the phone alarm or clocks as their medicine reminder tool. This is also consistent with findings from a qualitative study conducted in Lesotho that showed that mobile phone alarms and clocks are major facilitators of adherence among people living with HIV.^37^ Our findings build a case for the use of mobile phone technologies to enhance adherence to ART, as evidenced by the various studies.^38,39,40,41,42,43,44^

Our study also showed that old age was associated with ART adherence, a finding consistent with what has been found among the general population^18,36,45,46^ and other key populations.^47,48^ Although not examined in this study, it is possible that older FSWs have lived with HIV for a longer time and therefore have more ART experience than their younger counterparts. This finding suggests the need for targeted interventions to improve adherence among the young FSWs. Studies have shown that younger FSWs have unequal footing when negotiating in their communities. They are usually street-based, poorly educated, occupying low-skilled and poorly paying jobs, and are always in conflict with their older counterparts because of competition for clients.^49,50^

In addition, our study found lower odds of adherence to ART among FSWs who reported being widowed. This finding is consistent with results from a study among the general population of people living with HIV in Dakar, Senegal.^51^ Being widowed and a single mother who takes care of the children without a hand of the husband (many times originally the sole breadwinner), has been shown to be associated with depression.^52^ This, coupled with living in settings where FSWs are discriminated against and criminalised, could possibly affect adherence to ART.

Contrary to our expectation and evidence from other studies,^16,53,54^ data from our study did not show an association between alcohol use and adherence to ART. However, our finding is similar to that from a study among FSWs in India.^25^ Over half of the FSWs in our study reported alcohol use, similar to findings among FSWs in another study in Malawi^55^ and one in Uganda.^6^ Studies have documented the use of alcohol and other substances by FSWs as a way of coping with demands of their work.^56,57^ Our measurement of alcohol use was based on a Yes/No response to alcohol use in the last 3 months and not on the standard tool like audit score or the CAGE. This could partly explain this contradictory finding. Nonetheless, alcohol risk reduction counselling is necessary intervention for FSWs. Studies have indicated that alcohol consumption is not only associated with high-risk sexual behaviour^6,56^ but may also influence the survival of HIV-infected patients^58^ without altering the effectiveness of ART.^59^

## Conclusion and recommendations

We found a relatively low proportion of FSWs achieving the desirable level of adherence to ART. Our findings build a case for the development and scale-up of targeted intervention strategies to increase in ART adherence among FSWs. Special consideration to young FSWs is highly recommended based on the findings of our study. Incorporation of mobile phone technology applications in the routine adherence counselling follow-up services should be explored further and embraced as appropriate. These could include adherence counselling, text message reminders, voice notes or even peer support information technology platform, especially to the young FSWs.
